# β-Amyloid Clustering around ASC Fibrils Boosts Its Toxicity in Microglia

**DOI:** 10.1016/j.celrep.2020.02.025

**Published:** 2020-03-17

**Authors:** Lea L. Friker, Hannah Scheiblich, Inga V. Hochheiser, Rebecca Brinkschulte, Dietmar Riedel, Eicke Latz, Matthias Geyer, Michael T. Heneka

**Affiliations:** 1 Department of Neurodegenerative Disease and Gerontopsychiatry/Neurology, University of Bonn Medical Center, 53127 Bonn, Germany; 2 German Center for Neurodegenerative Diseases (DZNE), 53127 Bonn, Germany; 3 Department of Infectious Diseases and Immunology, University of Massachusetts Medical School, Worcester, MA 01655, USA; 4 Institute of Structural Biology, University of Bonn, 53127 Bonn, Germany; 5 Max Planck Institute for Biophysical Chemistry, Department of Structural Dynamics, 37077 Göttingen, Germany; 6 Institute of Innate Immunity, University of Bonn, 53127 Bonn, Germany; 7 Lead Contact

## Abstract

Alzheimer’s disease is the world’s most common neurodegenerative disorder. It is associated with neuroinflammation involving activation of microglia by β-amyloid (Aβ) deposits. Based on previous studies showing apoptosis-associated speck-like protein containing a CARD (ASC) binding and cross-seeding extracellular Aβ, we investigate the propagation of ASC between primary microglia and the effects of ASC-Aβ composites on microglial inflammasomes and function. Indeed, ASC released by a pyroptotic cell can be functionally built into the neighboring microglia NOD-like receptor protein (NLRP3) inflammasome. Compared with protein-only application, exposure to ASC-Aβ composites amplifies the proinflammatory response, resulting in pyroptotic cell death, setting free functional ASC and inducing a feedforward stimulating vicious cycle. Clustering around ASC fibrils also compromises clearance of Aβ by microglia. Together, these data enable a closer look at the turning point from acute to chronic Aβ-related neuroinflammation through formation of ASC-Aβ composites.

## In Brief

Friker et al. investigate the reaction of primary microglia to exogenous ASC and ASC-Aβ composites. They uncover a vicious circle involving amplified NLRP3 inflammasome activity and reduced Aβ clearance in the presence of ASC that might play a key role in Alzheimer’s disease progression.

## Graphical Abstract

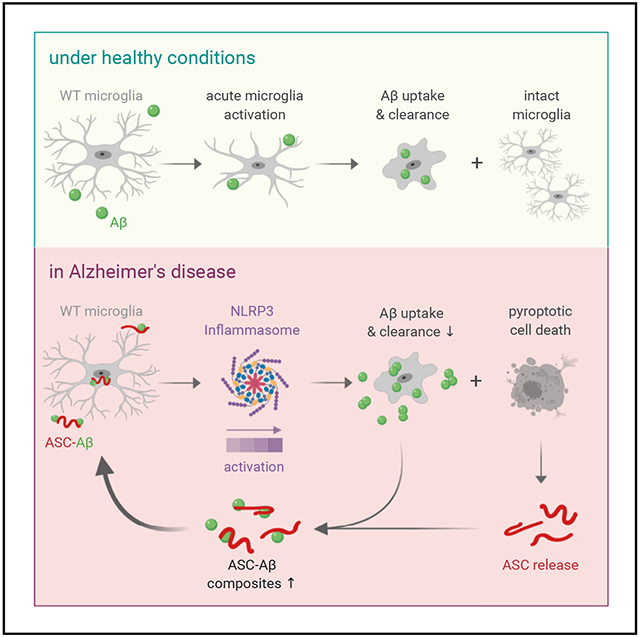

## INTRODUCTION

Alzheimer’s disease (AD) is the most common dementing illness, characterized by progressive memory decline and cognitive dysfunction (Zvĕřová, 2019). The pathological hallmarks of AD are deposition of extracellular β-amyloid (Aβ) and intraneuronal aggregation of neurofibrillary tangles composed of the microtubule-associated protein tau (Nelson et al., 2012). Under healthy conditions, the balance of Aβ and tau deposition and clearance is maintained by brain-resident microglia. However, in the AD brain, this equilibrium is shifted toward protein deposition (Sarlus and Heneka 2017; Heneka et al. 2015).

As resident immune effector cells of the CNS, microglia play a crucial role in mediating brain homeostasis and the innate immune response against a wide range of pathogenic factors (Clayton et al., 2017; Hanisch and Kettenmann, 2007). Microglia sense a variety of microbial molecules called pathogen-associated molecular patterns (PAMPs) but also host-derived danger-associated molecular patterns (DAMPs) by pattern recognition receptors (PRRs). PRR ligation then fuels signaling transduction pathways that induce an inflammatory response and also lead to clearance of debris by phagocytosis (Heneka et al., 2014). Recognition of oligomeric of fibrillar Aβ, which either serves as a PAMP or a DAMP (Terrill-Usery et al., 2014), rapidly triggers NLRP3 inflammasome activation (Halle et al., 2008; Kinney et al., 2018). Furthermore, NLRP3 inflammasome activation relies on two signals: transcriptional upregulation of inflammasome components via the transcription factor nuclear factor κB (NF-κB) (Kawai and Akira, 2010) and a second signal generated by DAMP-induced ion fluxes, mitochondrial reactive oxygen species (ROS) production, or lysosomal destabilization, which, in turn, leads to assembly and activation of the inflammasome (Yang et al., 2019).

The NLRP3 inflammasome is a multiprotein complex in which the receptor protein NLRP3 is bridged to the zymogen pro-caspase-1 via the adaptor protein apoptosis-associated speck-like protein containing a CARD (ASC). Upon dimerization, caspase-1 becomes activated by auto-processing, which, in turn, leads to cleavage of pro-inflammatory cytokines such as pro-interleukin-1β (pro-IL-1β) and pro-IL-18 (Boucher et al., 2018). In addition, caspase-1 cleaves Gasdermin D (GSDMD), a pyroptosis executioner protein (Bergsbaken et al., 2009), resulting in formation of pores in the plasma membrane and leading to cell lysis because of ion flux and subsequent cytosolic swelling (Liu et al., 2016). Moreover, GSDMD-induced pore-formation results in release of IL-1β into the extracellular space (Rathinam et al., 2019).

The adaptor protein ASC is composed of a N-terminal pyrin domain (PYD) and a C-terminal caspase recruitment domain (CARD) (Franklin et al., 2018). Homotypic intramolecular PYD-PYD interactions initiate formation of a helical filament, which allows intermolecular CARD-CARD interactions with the CARD domain of pro-caspase-1, causing its activation to form mature caspase-1 (Fernandes-Alnemri et al., 2007). Moreover, it has been demonstrated previously that ASC accumulates in cell-free supernatants of inflammasome-activated macrophages and build up ASC aggregates called ASC specks. Extracellular ASC specks have the ability to recruit and activate pro-caspase-1 and pro-IL-1β in cell cytosol or cell-free supernatant, further referred to as “prion-like” activities of ASC (Franklin et al., 2014). In Alzheimer’s pathology, rapidly after being released, Aβ_1–42_ binds ASC specks and accelerates its oligomerization, indicating cross-seeding activity of ASC in Aβ aggregation (Venegas et al., 2017).

Here we aimed to investigate the effects of ASC-Aβ composites on NLRP3 inflammasome activation in primary mouse microglia. We show that exogenous ASC induces ASC-dependent mechanisms in otherwise ASC-deficient microglia. Moreover, ASC-Aβ composites amplified NLRP3 inflammasome activation in comparison with ASC only or Aβ, resulting in pyroptotic cell death. In addition, we show that Aβ clearance by microglia is impaired in the presence of extracellular ASC aggregates, suggesting a possible mechanism involved in Aβ deposition in AD. Taken together, our findings indicate that Aβ clustering around ASC fibrils boosts its toxicity in microglia.

## RESULTS

**Figure 1.**
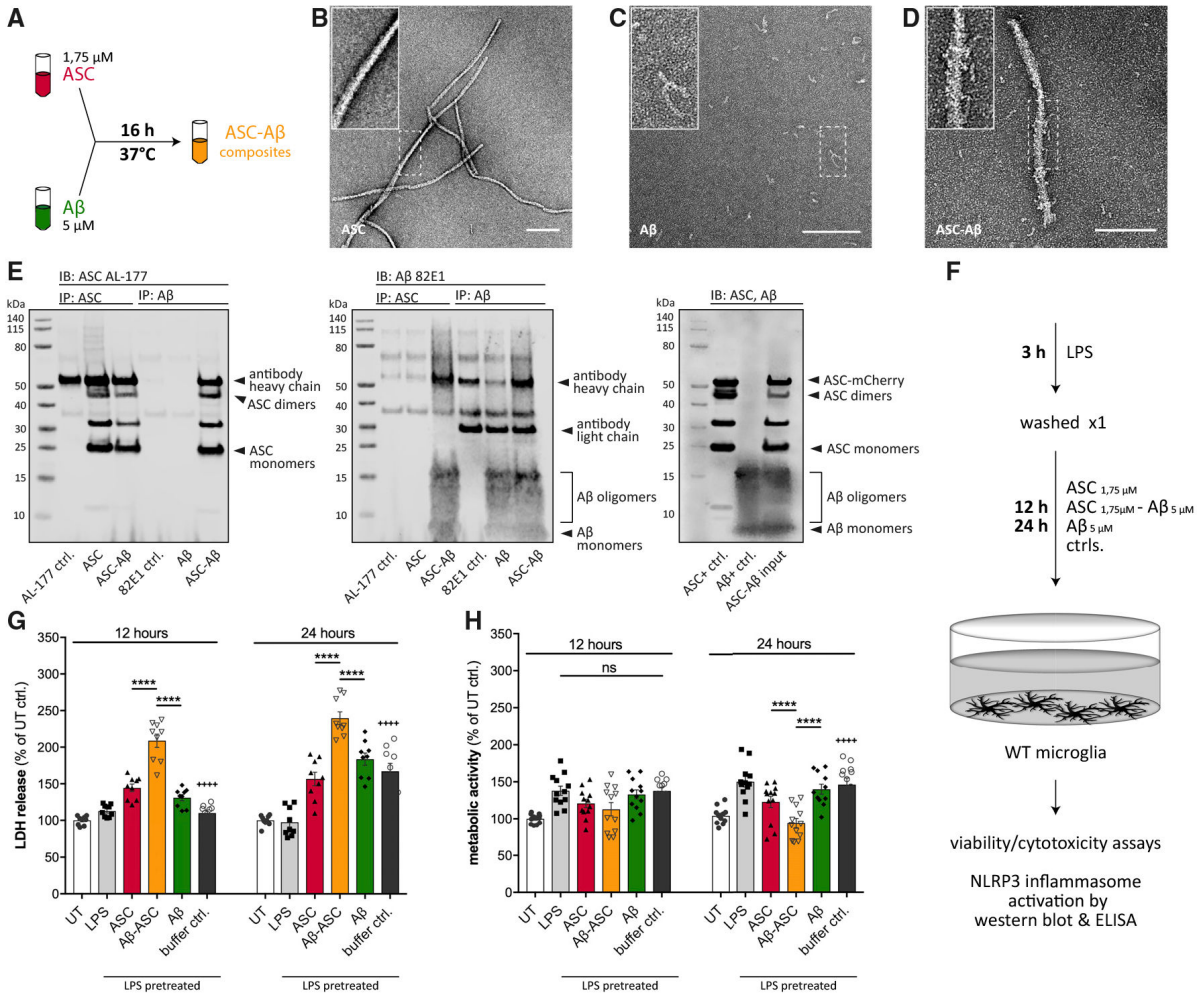
Aβ Clusters around ASC Fibrils, Forming ASC-Aβ Composites that Induce Cell Death (A) Schematic drawing of the ASC-Aβ composite-building protocol. (B–D) EM of ASC fibrils (B), Aβ (C), and ASC-Aβ composites (D). (E) coIP of pre-incubated ASC and Aβ, confirming binding of ASC to Aβ and formation of composites. Left: immunoblot (IB), ASC (AL-177). Center: IB, Aβ (82E1). Right: IB input control. (F) Schematic drawing of the experimental setup used in this study. (G and H) LDH release (G) and metabolic activity (H) measurements after 12 and 24 h of treatment with different components. Data were collected from three independent experiments (n = 3) with three technical replicates per assay (N = 9). All graphs are presented as mean ± SEM and were analyzed by two-way ANOVA followed by Tukey’s multiple comparisons test. Levels of significance are indicated as follows: *p < 0.05, **p < 0.01, ***p < 0.001, ****p < 0.0001. Asterisks indicate significance between groups connected by lines; plus symbols indicate significance between ASC-Aβ composites and volume-equal buffer control-treated groups. Scale bars, 200 nm (B–D).

Aβ monomers have been shown to associate and cluster onto ASC specks in the extracellular space (Venegas et al., 2017). To reproduce previous findings in our experimental setup, we co-incubated ASC with an about 3-fold molar excess of Aβ in serum-free medium or buffer (Figure 1A). Binding of both proteins was confirmed by transmission electron microscopy (TEM) (Figures 1B-1D) and co-immunoprecipitation (coIP) (Figure 1E).

In electron microscopy (EM), ASC was seen to arrange into long, slightly twisted helical filaments showing a uniform surface (Figure 1B). In contrast, Aβ formed oligomeric complexes (Figure 1C) that were found in close association with ASC fibrils when co-incubated (Figure 1D). These data were confirmed by coIP analysis using anti-ASC and anti-Aβ specific antibodies, respectively, indicating interaction of ASC and Aβ in a cell-free environment (Figure 1E).

To analyze the effect of these ASC-Aβ composites on cell survival in primary microglia, lipopolysaccharide (LPS)-primed cells were treated with ASC_1.75μM_, Aβ_5μM_, or ASC_1.75μM_-Aβ_5μM_ composites for 12 h and 24 h, respectively (Figure 1F). Release of lactate dehydrogenase (LDH) in response to ASC-Aβ treatment was used as an indicator of cell death (Giordano et al., 2011). Irrespective of LPS priming, microglia exposed to ASC-Aβ composites showed a significant increase in LDH release compared with ASC or Aβ alone (Figure 1G). Interestingly, the metabolic activity in microglia treated with ASC-Aβ composites did not change within 12 h but was significantly reduced after 24 h compared with ASC- or Aβ-treated cells (Figure 1H).

### ASC-Aβ Composites Activate the NLRP3 Inflammasome and Induce Pyroptotic Cell Death in Microglia

**Figure 2.**
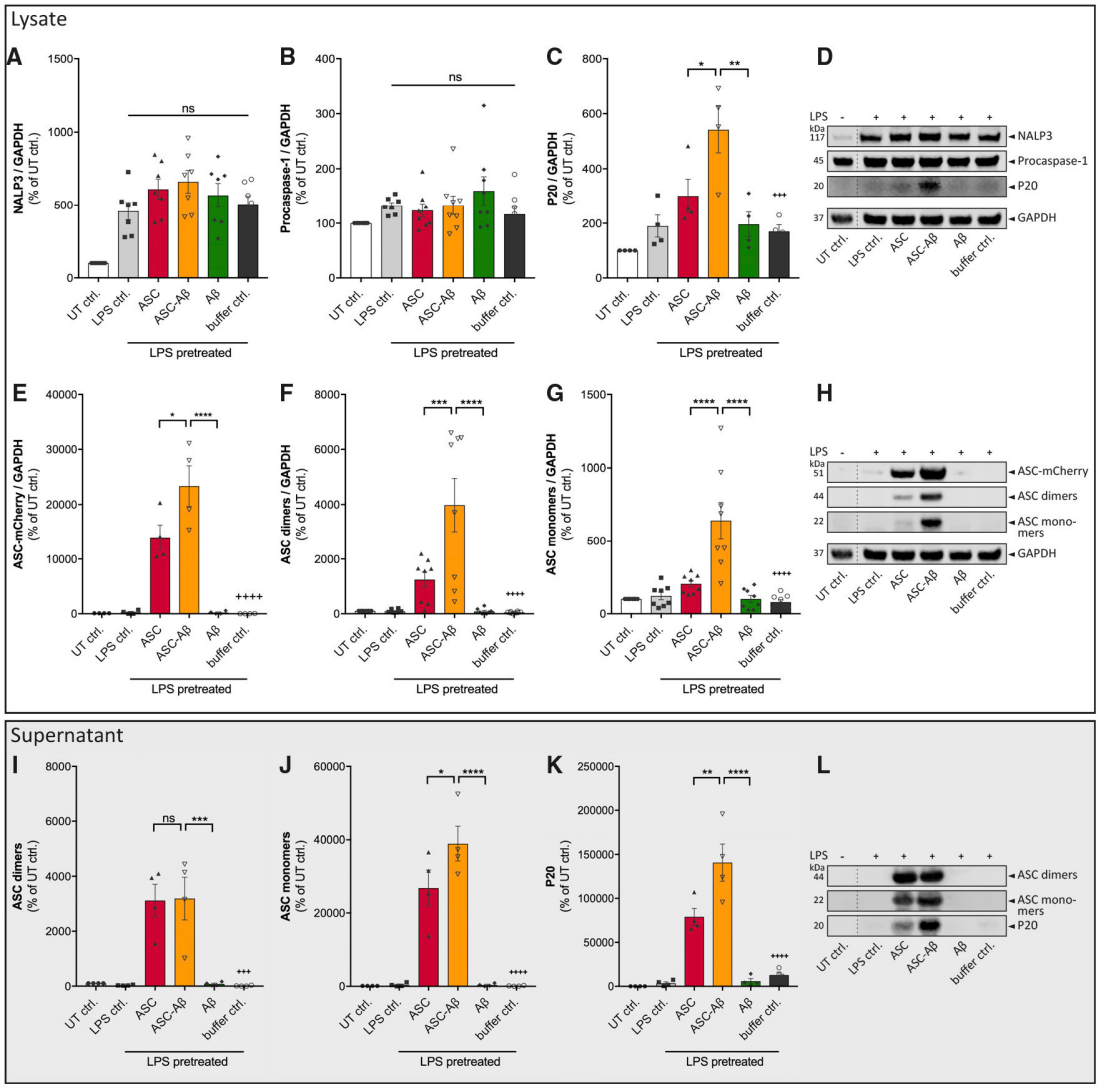
ASC-Aβ Composites Induce Caspase-1 Cleavage to a Greater Extent Than ASC or Aβ Alone (A–C and E–G) IB analysis and quantification of cell lysates of primary WT microglia primed for 3 h with 100 ng/mL LPS and exposed to ASC, Aβ, or ASC-Aβ composites for 12 h. Blots of cell lysates of primary WT microglia (D and H) were stained for NLRP3 (A), caspase-1 (B and C), different conformations of ASC (E–G), and GAPDH. (I–K) IB analysis using precipitated supernatants (L)were stained for ASC dimers (I), ASC monomers (J), and cleaved caspase-1 subunit p20 (K). Empty wells and wells containing experimental samples that are not part of this study are not displayed. Vertical lines in blots indicate spliced sections (D, H, and L). For better reader convenience, the GAPDH signal, which belongs to the same original blot, has been separated in (D) and (H). Data were collected from four (C, E, I, J, and K) or eight (A, B, F, and G) independent experiments (n = 4 or 8). All graphs are presented as mean ± SEM and were analyzed by one-way ANOVA in conjunction with Tukey’s test (A, B, E–G, and I–K) and Kruskal-Wallis test combined with a Dunn’s post hoc test (C). Levels of significance are indicated as follows: *p < 0.05, **p < 0.01, ***p < 0.001, ****p < 0.0001. Asterisks indicate significance between groups connected by lines; plus symbols indicate significance between ASC-Aβ composites and volume-equal buffer control-treated groups. See also Figure S1.

To study whether the observed cell death of microglia in response to ASC-Aβ composite treatment originates from pyroptosis, we correlated NLRP3 inflammasome activation. As expected, LPS treatment of microglia induced priming of the inflammasome, as shown by an increased expression level of the receptor protein NLRP3 in the cell lysate, whereas the levels of pro-caspase-1 remained unaltered (Figures 2A, 2B, and 2D). Interestingly, caspase-1 cleavage was significantly increased in response to ASC-Aβ composite treatment in both cell lysates (Figures 2C and 2D) and supernatants (Figures 2K and 2L). Moreover, significantly higher levels of ASC were detected in cell lysates of the composite-treated group in comparison with ASC only, occurring with all detected ASC aggregates: ASC-mCherry, ASC dimers, and ASC monomers (Figures 2E-2H). In contrast, microglia supernatants showed higher levels of ASC monomer after composite treatment (Figures 2J and 2L), whereas dimers stayed constant (Figures 2I and 2L).

To discriminate between cell-derived and recombinant human ASC used for cell treatment, a mouse-specific anti-ASC antibody was applied to visualize ASC aggregation and speck release into the extracellular environment (Figure S1). In addition to ASC speck formation, we observed morphological changes in microglia in the course of ASC speck formation. ASC-expressing microglia displayed a ramified shape, whereas microglia retracted their processes, resulting in a more activated phenotype during ASC speck formation. ASC speck-releasing cells were further found to undergo transformation, resulting in a highly activated phenotype, potentially indicating pyroptosis.

**Figure 3.**
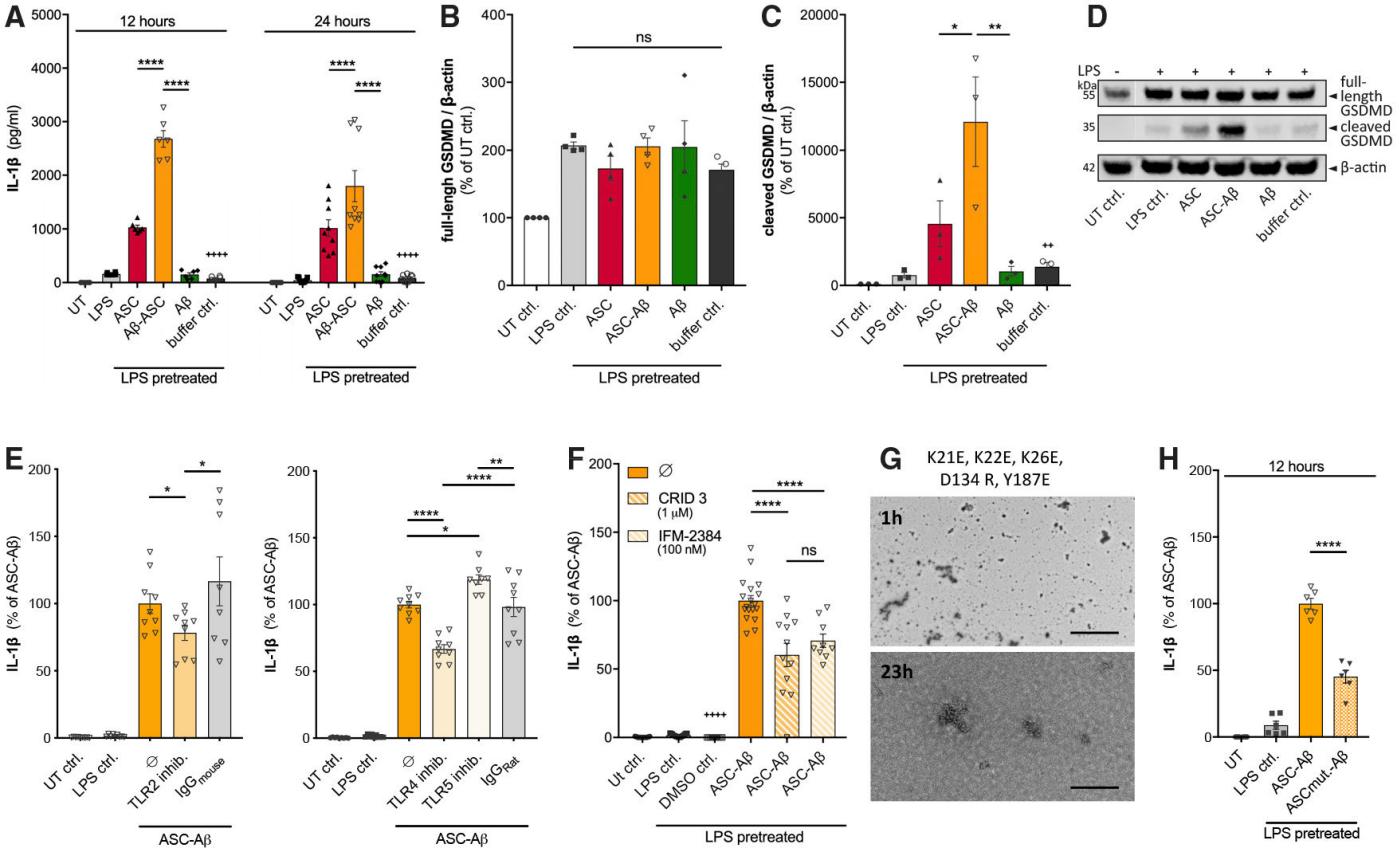
ASC-Aβ Composites Induce IL-1β by TLR2 and TLR4 Ligation, a Process Dependent on ASC’s Fibrillation Ability (A) IL-1β levels in conditioned medium of primary microglia after 12 and 24 h of exposure to ASC, Aβ, or ASC-Aβ composites (n = 3 independent experiments with triplicate treatments for all conditions). (B–D) IB analysis and quantification of full-length Gasdermin D (GSDMD; n = 3; B and D) and cleaved NTD (n = 4; C and D). Empty wells and wells containing experimental samples, which are not part of this study are not displayed. Vertical lines in blots indicate spliced sections (D). (E) IL-1β levels in conditioned medium of primary microglia treated for 12 h with TLR2-, TLR4-, and TLR5-neutralizing antibodies as well as the respective IgG isotype controls in parallel to stimulation with ASC, Aβ, or ASC-Aβ composites (n = 3 independent experiments with triplicate treatments for all conditions). (F) IL-1β levels in conditioned medium of primary microglia after NALP3 inflammasome inhibition using CRID3 or IFM-2384 (n = 3 independent experiments with triplicate treatments for all conditions). (G) EM of ASC carrying PYD (K21E, K22E, and K26E) and CARD (D134R and Y187E) mutations after 1 and 23 h of incubation at 37°C. (H) IL-1β levels in conditioned medium of primary microglia treated with ASC-Aβ composites or mutated ASC pre-incubated with Aβ (n = 3 independent experiments with duplicate treatments for all conditions). All graphs are presented as mean ± SEM and were analyzed by two-way ANOVA (A) or one-way ANOVA (B–H) in conjunction with Tukey’s test. Levels of significance are indicated as follows: *p < 0.05, **p < 0.01, ***p < 0.001, ****p < 0.0001. Asterisks indicate significance between groups connected by lines; plus symbol indicate significance between ASC-Aβ composites and volume-equal buffer control-treated groups. Scale bar, 2 μm (G, upper panel) and 200 nm (G, lower panel). See also Figures S1 and S2.

To monitor the release of IL-1β into the extracellular space as a consequence of ASC speck formation and subsequent caspase-1 maturation, we performed ELISA experiments after 12 and 24 h of treatment (Figure 3A). ASC only as well as ASC-Aβ composites led to a significant increase in IL-1β release after both time points compared with Aβ alone. Interestingly, ASC-Aβ composites significantly augmented IL-1β levels compared with ASC only (~2.6-fold after 12 h and ~1.8-fold after 24 h). The highest absolute IL-1β levels were determined after 12 h of treatment; hence, the following experiments were performed using this time point only.

Besides cleavage of pro-IL1β and pro-IL-18, caspase-1 also cleaves GSDMD, which subsequently forms GSDMD pores that facilitate secretion of mature IL-1β into the supernatant (Rathinam et al., 2019). To monitor the effect of ASC-Aβ composites on cleavage of GSDMD, the levels of full-length and amino-terminal GSDMD (NTD) in response to the respective treatment were determined by western blotting (Figures 3B-3D). Interestingly, treating the cell with ASC-Aβ composites largely induced GSDMD cleavage in microglia (Figures 3C and 3D), which is in line with the IL-1β release we detected by ELISA readings (Figure 3A).

To elucidate the underlying mechanism by which ASC-Aβ composites activate the NLRP3 inflammasome, we tested for involvement of Toll-like receptors (TLRs) because these receptors have been found to play a key role in neuroinflammation (Kumar, 2019). To analyze the effect of TLR2, TLR4, and TLR5, release of IL-1β was determined in LPS-primed microglia exposed to ASC-Aβ composites in the presence or absence of TLR-neutralizing antibodies (Figure 3E). Here we show that TLR2 and TLR4 neutralization significantly decreased release of IL-1β. In contrast, inhibition of TLR5 slightly increased IL-1β levels in the supernatant. The corresponding immunoglobulin G (IgG) isotype controls did not have any effect.

To determine whether release of IL-1β in response to ASC-Aβ composites is NLRP3 dependent, we again co-treated microglia with ASC-Aβ composites and the specific NLRP3 inhibitors CRID3 (MCC950) and IFM-2384 (Figure 3F). As expected, co-treatment with both inhibitors led to a significant reduction in IL-1β release by about 40%. To determine whether IL-1β (Figure 3F) was processed, we performed an immunoblot analysis of supernatants (Figure S2E). We found that CRID3 and IFM-2384 treatment decreased the release of mature IL-1β from microglia but detected continuously high amounts of pro-IL-1β in the supernatants. In addition, immunoblot analysis consistently detected an additional unconventional precursor form of pro-IL-1β at around 25 kDa (p25) besides the conventional 31 kDa (p31) form. These data confirm that high amounts of pro-IL-1β were released and detected by our ELISAs.

### IL-1β Release Induced by ASC-Aβ Composites Depends on ASC’s Fibrillation Ability

To determine whether activation of the NLRP3 inflammasome and the subsequent increase in IL-1β release is dependent on the fibrillation potential of ASC, we generated an ASC variant incapable of filament formation. Indeed, TEM revealed that mutant ASC carrying three mutations within the PYD interface (K21E, K22E, and K26E) and two mutations within the CARD domain (D134R and Y187E) lacks filament formation (Figure 3G). Interestingly, ASC-Aβ composites containing mutated ASC induced only half of the IL-1β release measured after treating the cells with non-mutated ASC-Aβ composites (Figure 3H).

### Exogenous ASC Induces Caspase-1 Cleavage and IL-1β Release in ASC-Deficient Microglia

**Figure 4.**
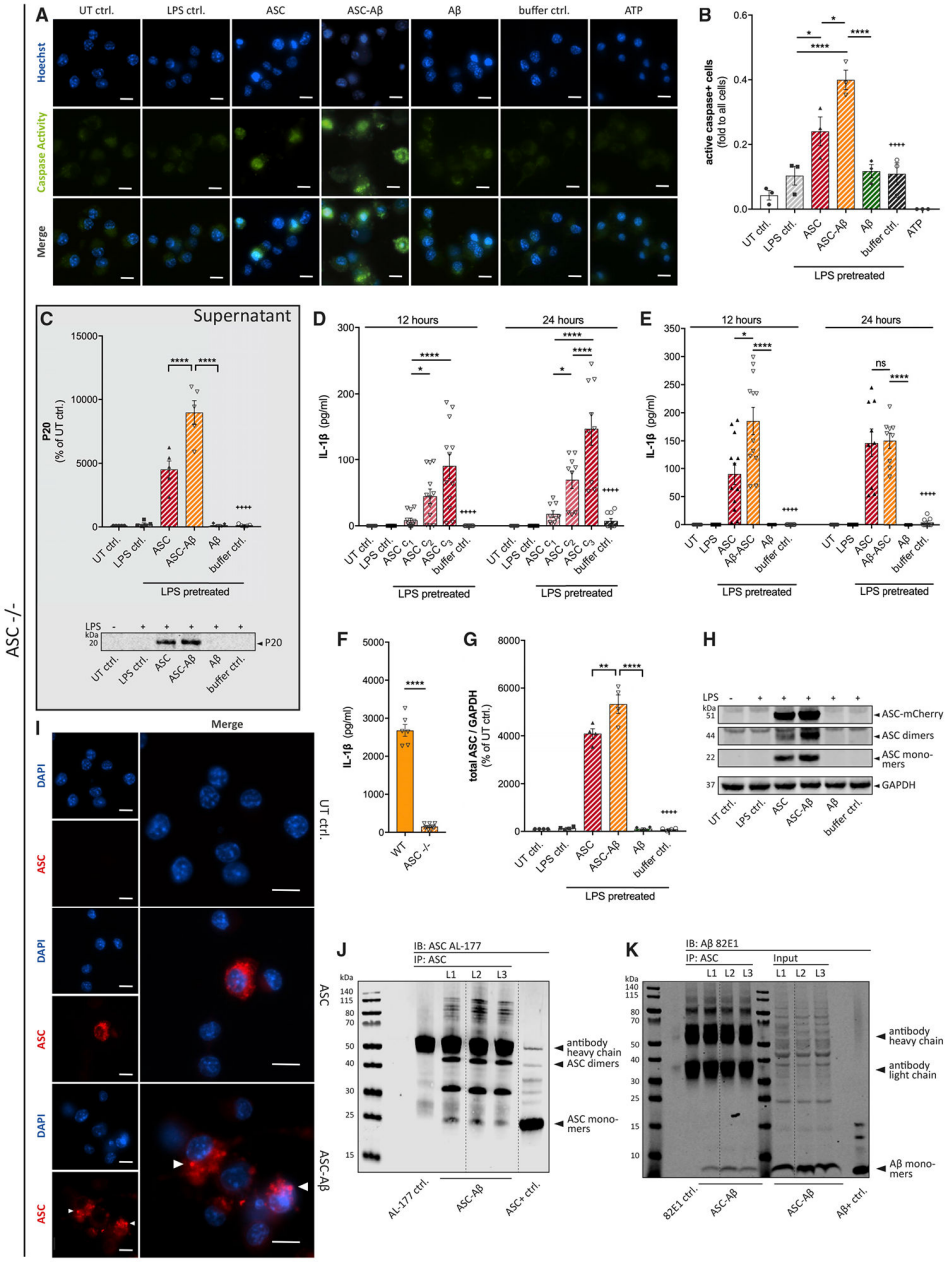
Exogenous ASC Can Replace Endogenous ASC in ASC-Deficient Cells and Induce ASC-Dependent Signaling Pathways (A and B) Immunocytochemical detection (A) and quantification (B) of caspase-1 activity in ASC-deficient macrophages treated with or without exogenous ASC. (C) The caspase-1 subunit p20 was detected by IB analysis in precipitated supernatants from ASC-deficient microglia. (D–F) IL-1β levels in conditioned medium of ASC-deficient primary microglia after exposure to different concentrations of exogenous ASC: c_1_ = 0.22 μM, c_2_ = 0.66 μM, c_3_ = 1.75 μM (D); IL-1β levels in conditioned medium of ASC-deficient primary microglia treated with ASC, Aβ, or ASC-Aβ composites for 12 and 24 h (E); and IL-1β levels in conditioned medium of primary WT microglia and ASC-deficient microglia treated with ASC-Aβ composites for 12 h (F). (G and H) IB analysis and quantification (G) of ASC monomers, dimers, and ASC-mCherry in ASC-deficient microglia cell lysates (H) (n = 4). (I) Immunostaining of ASC-deficient macrophages treated with exogenous ASC and stained for ASC internalization and ASC speck formation after ASC-Aβ composites treatment (arrowheads). (J and K) CoIP of ASC and Aβ in ASC-deficient microglia cell lysates. IP: ASC. IB: ASC (AL-177) (J), IB: Aβ (82E1) and input controls (K). Empty wells and wells containing a lysate control negative for both proteins are not displayed. Vertical lines in blots indicate spliced sections. Data were collected from three independent experiments (n = 3) with three technical replicates perassay (N = 9) (D–F). All graphs are presented as mean ± SEM and were analyzed by two-way ANOVA (D and E), one-way ANOVA in conjunction with Tukey’s test (B, C, and G), or unpaired t test (F). Levels of significance are indicated as follows: *p < 0.05, **p < 0.01, ***p < 0.001, ****p < 0.0001. Asterisks indicate significance between groups connected by lines; plus symbols indicate significance between ASC-Aβ composites and volume-equal buffer control-treated groups. Images were taken at 40× magnification. Scale bars, 10 μm (A and I).

ASC is known to possess prion-like activity as it propagates from a pyroptotic macrophage to another recipient cell while preserving its activity. Moreover, accumulation of extracellular ASC specks has been shown to induce IL-1β maturation in bone marrow-derived macrophages (Franklin et al., 2014). To investigate whether similar prion-like activity of ASC can be observed in microglia, we treated ASC-deficient macrophages and ASC-deficient primary microglia with exogenously applied recombinant ASC (Figure 4). To mimic a second stimulus for NLRP3 inflammasome activation, adenosine triphosphate (ATP) was used as a positive control (Karmakar et al., 2016). As expected, ATP induced caspase-1 activation in wild-type (WT) cells (data not shown) but not in ASC-deficient macrophages (Figures 4A and 4B). Interestingly, exogenously applied ASC largely induced caspase-1 activity. In line with our data, we detected the highest levels of caspase-1 activity in macrophages that were exposed to ASC-Aβ composites (Figure 3B). Using ASC-deficient microglia, we measured significantly increased levels of the auto-processed caspase-1 fragment p20 in cell supernatants of cells treated with ASC and ASC-Aβ composites (Figure 4C). Again, treatment with ASC-Aβ composites amplified caspase-1 cleavage by about 2-fold compared with ASC treatment alone.

Most importantly, exogenously applied ASC induced IL-1β release in ASC-deficient microglia in a dose- and time-dependent manner (Figure 4D). After 12 h, ASC-Aβ composites elevated IL-1β release nearly 2-fold compared with ASC only. However, there was no significant difference remaining after 24 h (Figure 4E). When comparing the total extent of IL-1β measured in ASC-deficient versus WT microglia, is noticeable that the tendencies of differently treated groups are similar in both genotypes, although total IL-1β levels are reduced to approximately 10% in ASC-deficient cells (Figure 4F).

As expected, cell lysates of ASC-deficient microglia exposed to exogenous ASC showed high total ASC levels (Figures 4G and 4H). Interestingly, microglia treated with ASC-Aβ composites contained significantly more ASC compared with ASC-only-treated cells.

Next we aimed to investigate whether exogenously applied ASC, when taken up by ASC-deficient cells, still possesses the ability to form intracellular ASC specks. Indeed, exposure of macrophages to ASC increased the number of ASC-positive cells, which was further enhanced in the ASC-Aβ composite-treated group (Figure 4I). Moreover, by visualization of internalized ASC, we saw that speck formation was further amplified when cells were treated with ASC-Aβ composites (Figure 4I, bottom panel).

The interaction of ASC and Aβ in cell lysates of ASC-deficient microglia was still preserved, as shown in coIP experiments (Figures 4J and 4K). Interestingly, higher quantities of ASC dimers than ASC monomers were detected in lysates of cells treated with ASC-Aβ composites (Figure 4J).

### ASC Binding Exacerbates Uptake of Aβ and Decelerates Its Degradation

**Figure 5.**
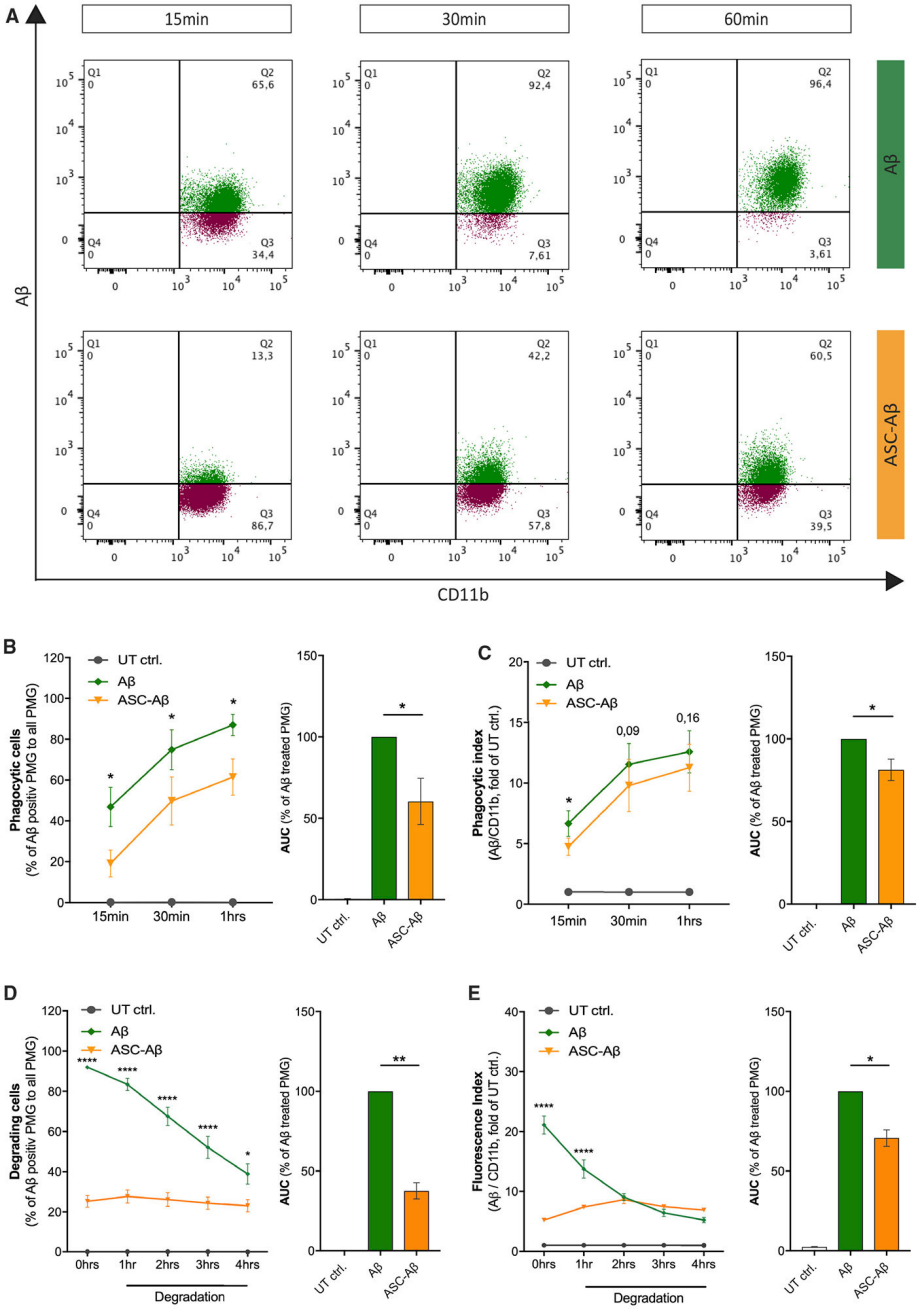
Aβ Clearance by Microglia Is Affected When Bound to ASC (A) Representative FACS graphics demonstrating the engulfment of Aβ by microglia upon treatment with Aβ only or ASC-Aβ composites for 15, 30, and 60 min. (B) Quantification of microglia engulfing Aβ over time. (C) Phagocytic index of microglia engulfing Aβ over time. (D and E) Quantification and comparison of relative Aβ degradation by WT microglia over 1, 2, 3, and 4 h in the presence or absence of ASC, measured after 1 h of Aβ phagocytosis. (D) Quantification of Aβ-positive microglia over time. (E) Fluorescence index of microglia degrading Aβ over time. Data were collected from three independent experiments (n = 3) with two technical replicates per assay (N = 6). All graphs are presented as mean ± SEM and were analyzed by paired t test for phagocytic cells and index (B and C) as well as two-way ANOVA followed by Tukey’s multiple comparisons test for degradation analysis (D and E) and unpaired t test for area under the curve (AUC; percentage of Aβ-treated primary microglia [PMG]; B–E). Levels of significance are indicated as follows: *p < 0.05, **p < 0.01, ***p < 0.001, ****p < 0.0001. See also Figure S2.

Microglia play an outstanding role in amyloid clearance in the healthy brain but also during AD progression (Baik et al., 2016; Cho et al., 2014). Thus, we analyzed the potential effect of ASC-Aβ binding on phagocytic clearance of Aβ by primary microglia. To quantify the engulfment of Aβ, we treated microglia either with fluorescein (FAM)-labeled Aβ or ASC-FAM-Aβ composites and assessed the uptake of Aβ using fluorescence-activated cell sorting (FACS) analysis (Figure S2A). Interestingly, in the presence of ASC-Aβ composites, uptake of Aβ was decreased by about 35% after 1 h compared with primary microglia treated with Aβ only (Figures 5A-5C).

In addition to phagocytic uptake, we determined the degradation capacity of microglia to prove an imbalance in uptake and degradation. We found that microglia constantly degrade Aβ in a time-dependent manner with a degradation rate of about 20%–25% per hour (Figures 5D and 5E). In contrast, when microglia were exposed to ASC-FAM-Aβ composites, uptake was largely diminished, and degradation was fully blocked (Figures 5D and 5E). We confirmed these findings using immunoblot analysis of cell lysates collected after an allowed degradation time of 12 h (Figure S2B).

A common factor implicated in a number of cellular processes, including phagocytosis, proliferation, survival, and regulation of inflammatory cytokine production, is the receptor TREM2 (Ulrich and Holtzman, 2016). Consequently, we checked for changes in TREM2 expression levels by western blot (Figures S2C and S2D). Interestingly, TREM2 expression was decreased in cells treated with ASC-Aβ, indicative of a modulatory effect of ASC-Aβ composites on TREM2 activity. Our data indicate an ASC-Aβ-induced imbalance between uptake and degradation, resulting in deposition of cytotoxic protein accumulation intra- and extracellularly.

## DISCUSSION

The link between Aβ deposition and microglial dysfunction has already been studied extensively (Cai et al., 2014; Sarlus and Heneka, 2017). Unravelling the underlying pathway, we identified ASC as a key player (Venegas et al., 2017). Our previous study found expression of ASC to be increased in an Alzheimer’s disease mouse model compared with a WT control. Moreover, ASC was shown to rapidly interact with pathogenic Aβ_1–42_ extracellularly, suggested to underlie microglial dysfunction. Data showing a direct connection between ASC-Aβ composites leading to microglial dysfunction are still pending; hence, we aimed to unravel the resulting cellular effects on microglia exposed to Aβ-ASC composites. Indeed, our present study revealed severe toxicity emanating from ASC-Aβ composites compared ASC only or Aβ. Microglia exposed to ASC-Aβ subsequently underwent cell death, displaying increased LDH release (Figure 1G) and reduced metabolic activity (Figure 1H). In addition, composite-treated microglia showed ASC speckling (Figure S1), caspase-1 cleavage (Figures 2C and 2D) and release of its active fraction (Figures 2K and 2L), pro-inflammatory cytokine maturation (Figure 3A), as well as highly elevated levels of the pore-forming NTD (Figures 3C and 3D). Thus, we considered the observed cell death to be pyroptotic. Caspase-1 activity has been shown to accelerate IL-1β secretion via rapid GSDMD-dependent pathways (Monteleone et al., 2018). This mechanism could possibly also underlie the pyroptotic cell fate detected here.

To confirm NLRP3 dependency of the observed IL-1β release, we used different modulators to inhibit the NLRP3-initiated ASC assembly. The resulting total IL-1β levels were reduced by approximately 40% (Figure 3F), possibly because of decreased cleavage and impaired release of its mature form (Figure S2E), also considering further ASC-dependent inflammasomes such as the AIM or NLRC4 inflammasome (Freeman et al., 2017) to mediate generation of the remaining IL-1β levels.

Mutagenesis studies showed that clustering of ASC^PYD^ filaments and their condensation into ASC specks is mediated by the ASC^CARD^ exposed to the surface of the ASC^PYD^-initiated filament (Hoss et al., 2017). Furthermore, filament formation served as an amplification mechanism in inflammasome signaling, resulting in cytokine maturation (Dick et al., 2016). By mutating D134 and Y187 within the ASC^card^ domain, filament formation of the CARD domain only is almost completely disrupted (Li et al., 2018). Particular mutations of the type I interface mediating PYD-PYD interactions, such as K21Q, K21E/K22E, and K26E, also abolish formation of the ASC^PYD^ filament (Lu et al., 2014). Recently, we showed that full-length human ASC carrying three K-to-A mutations within the above mentioned PYD-PYD interface (K21A, K22A, and K26A) does not cross-seed Aβ_1–40_ aggregation (Venegas et al., 2017). Using an ASC variant deficient in PYD as well as CARD-mediated filament formation (K21E, K22E, K26E, D134 R, and Y187E), release of proinflammatory cytokines decreased tremendously, suggesting that the filamentary structure of ASC is mandatory for NLRP3 inflammasome activation in response to ASC-Aβ composites (Figures 3G and 3H).

Here we observed that exogenous ASC induces speck formation of cell-intrinsic ASC in WT microglia (Figure S1) and itself formed speck-like aggregates in the cytosol of ASC-deficient cells (Figure 4I). These findings underline the importance of ASC aggregation in inducing the NLRP3 inflammasome pathway (Latz et al., 2013; Stutz et al., 2013). ASC specks have been identified previously as an endogenous danger signal because injection of ASC specks caused acute inflammatory reactions in WT mice (Franklin et al., 2014). *In vitro*, ASC specks were shown to accumulated in the extracellular space but still retained the ability to mature pro-IL-1β. Moreover, it was shown that phagocytosed ASC specks still induced lysosomal damage and IL-1β production in macrophages.

Activation of the NLRP3 inflammasome by ASC-Aβ composites is thought to be transferred via a still unknown mediator. Because the expression of multiple TLRs on the microglia surface increases with the presence of pathogens or other pro-inflammatory stimuli (Olson and Miller, 2004), we examined the effect of TLRs. Inhibiting TLR2 and TLR4 significantly decreased release of IL-1β in response to ASC-Aβ treatment (Figure 3E), supporting the commonly accepted knowledge of Aβ being a target of both cell surface receptors (Letiembre et al., 2009) and inducing NLRP3 inflammasome activation (Liu et al., 2012; Walter et al., 2007).

It is speculated that, besides the interaction of Aβ with TLR2 and TLR4, multiple cell surface receptors could also be targeted, including CD36 and TLR6 (Doens and Fernández, 2014; Sheedy et al., 2013; Stewart et al., 2010). Aβ clustering around the ASC fibril might enable Aβ to interact with various cell surface receptors simultaneously, presumably boosting intracellular inflammatory cascade activation, revealing the toxicity of ASC-Aβ composites.

It has been shown recently that the soluble TLR5 Fc-fragment binds to oligomeric as well as fibrillar Aβ with high affinity and blocks its toxicity. Moreover, Aβ has been shown to modulate flagellin-mediated activation but does not by itself activate TLR5 signaling (Chakrabarty et al., 2018). Supporting the hypothesis of a protective role of TLR5, we showed that an increase in IL-1β levels in response to ASC-Aβ exposure is determined only in presence of a specific TLR5 inhibitor (Figure 3E).

Here, we present evidence that exogenous ASC has the ability to induce caspase-1 cleavage (Figures 4A-4C) and IL-1β maturation (Figures 4D and 4E) not only in peripheral macrophages but also in microglia, using an ASC-deficient genotype. ASC-Aβ composites induced higher p20 and IL-1β levels than ASC alone, showing the same tendencies as in the WT but reaching lower absolute values (Figure 4F), suggesting that cell-intrinsic ASC speck formation, which is also induced by ASC-Aβ composites (Figure S1), plays an important role in exogenous ASC-Aβ toxicity. Indeed, we detected higher levels of ASC in microglia lysates after ASC-Aβ composite treatment compared with ASC-only exposure, assuming that the increased uptake of ASC is composite mediated, which, in turn, leads to increased NLRP3 inflammasome activity (Figures 4G and 4H).

Of note, a stable interaction of ASC and Aβ is assumed because both proteins stay bound within the cell lysates (Figures 4J and 4K). Thus, it is hypothesized that Aβ stabilizes the ASC fibril intracellularly and possibly extends its time of availability and/or accelerates the pro-inflammatory cascade.

As described previously, missense variants in the TREM2 receptor are associated with a 2- to 4-fold increased risk of developing AD (Guerreiro et al., 2013; Jonsson et al., 2013). Moreover, it has been shown that exogenous expression of TREM2 in Chinese hamster ovary (CHO) or HEK293 cells increases phagocytic activity (Kleinberger et al., 2014; N’Diaye et al., 2009). Furthermore, TREM2 overexpression promotes clearance of Aβ_1–42_ by BV-2 cells and restored cell viability from Aβ-mediated neuroinflammation by downregulating TLRs (Long et al., 2019). However, inflammatory stimuli decrease TREM2 expression *in vitro* but increase TREM2 expression *in vivo* (Jay et al., 2017).

Our findings revealed increased pro-inflammatory cytokine levels (Figure 3A) in a TLR2-, TLR4-, and TLR5-dependent manner (Figure 3E). Moreover, a decrease in phagocytic activity (Figures 5A-5C) as well as a reduction in TREM2 expression (Figures S2C and 2D) in response to exposure of ASC-Aβ composites suggests a connection between these findings. We could also show that clustering of microglia to ASC fibrils resulted in impaired Aβ uptake (Figures 5A-5C). Further studies might consider the effect of TREM1 and TREM2 on ASC-Aβ phagocytosis because there is growing evidence that their overexpression increases Aβ clearance by microglia (Jiang et al., 2016; Xiang et al., 2016). In addition, microglia exposed to ASC-Aβ composites lost their ability to degrade Aβ compared with microglia exposed to Aβ only (Figures 5D and 5E; Figure S2B). Aggregate size as well as the interaction of ASC and Aβ itself might underlie the altered phagocytosis. Altogether, these findings reveal a possible mechanism involved in AD progression because Aβ plaque clearance by microglia is essentially important in the disease context (Hansen et al., 2018; Sarlus and Heneka, 2017).

## Conclusions

**Figure 6.**
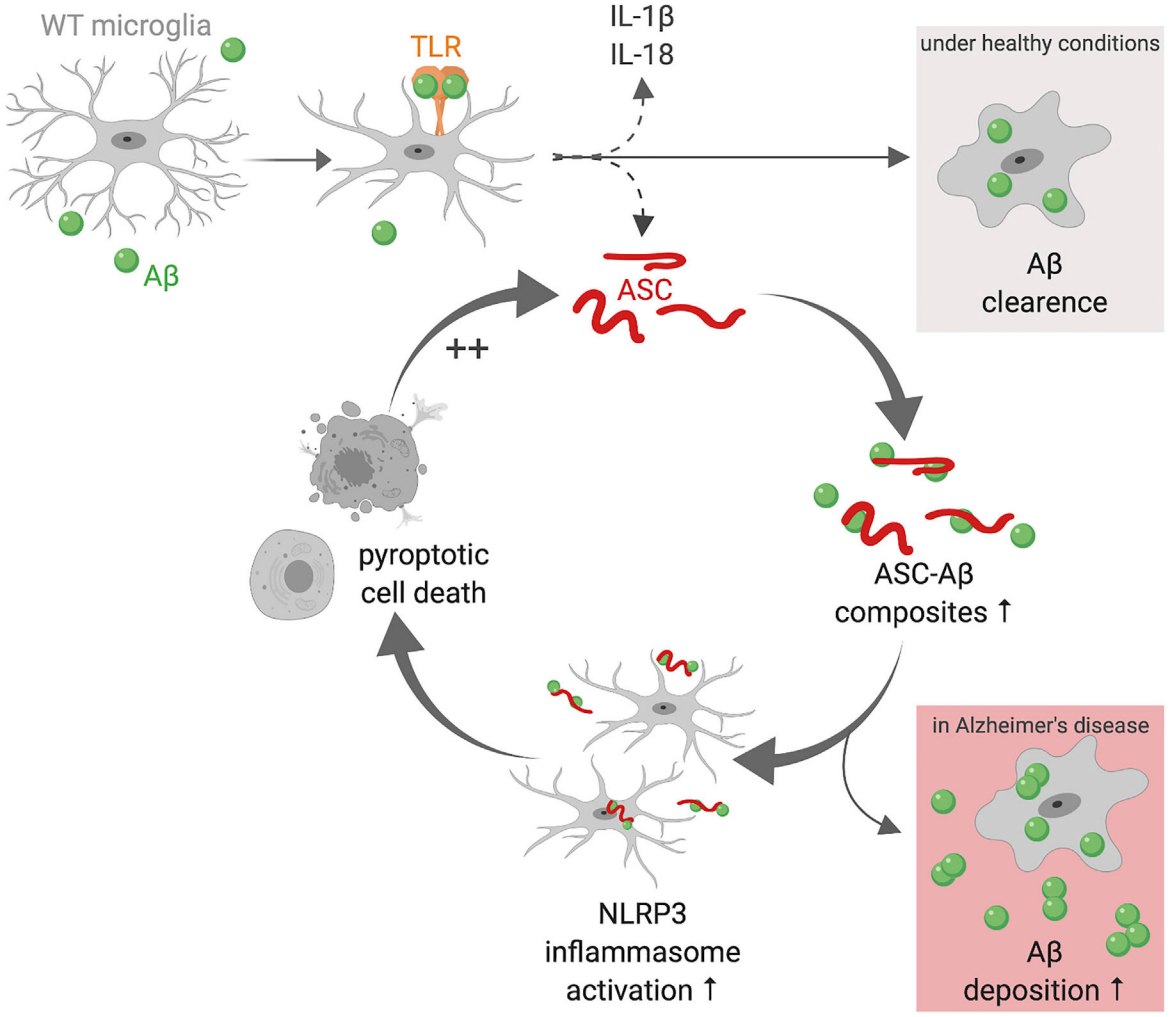
ASC-Aβ Binding Induces a Vicious Cycle in Microglia The schematic was created with BioRender (https://biorender.com). Aβ induces microglia activation via multiple cell surface receptors. Here we display TLRs as an example. Under healthy conditions, microglia activation leads to engulfment and clearance of Aβ. During microglia activation, NLRP3 inflammasome components, such as the adaptor protein ASC and the pro-inflammatory cytokines IL-1β and IL-18, are released to the extracellular space. There, Aβ clusters around ASC fibrils, resulting in ASC-Aβ composite formation. ASC-Aβ composites themselves boost NLRP3 inflammasome activation in the surrounding microglia, reducing microglia Aβ clearance ability and resulting in pyroptotic cell death. During pyroptosis, vast quantities of ASC are set free, starting the vicious cycle of ASC-Aβ composites all over. In the AD brain, this might be a reason for increased Aβ deposition.

Taken together, ASC contributes prion-like activities in microglia as in macrophages. Cells undergoing pyroptosis set free fully functional ASC that can be built into the NLRP3 inflammasome of the recipient cell. Even ASC-deficient cells were shown to induce caspase-1 cleavage and pro-inflammatory cytokine maturation mediated by exogenous ASC. In WT microglia, ASC induced cell-intrinsic ASC speck formation and release. Furthermore, exogenous ASC itself also formed speck-like aggregates inside the cell. Clustering of Aβ onto ASC fibrils led to multiple cellular responses, such as an increase in caspase-1 activation, IL-1β maturation, and cleavage of GSDMD. Moreover, ASC specks were found to be formed faster, and ASC was taken up in higher quantities. ASC and Aβ remained bound in the cell lysates, assuming Aβ to stabilize the ASC fibril and thereby boosting its toxicity. Thus, cells exposed to ASC-Aβ subsequently underwent pyroptosis, setting free ASC and leading to activation of the surrounding cells, inducing a vicious circle (Figure 6). Furthermore, ASC-Aβ binding was shown to prevent Aβ clearance by microglia *in vitro*, which might play a role in AD progression *in vivo*.

## STAR★METHODS

### LEAD CONTACT AND MATERIALS AVAILABILITY

Further information and requests for resources and reagents should be directed to and will be fulfilled by the Lead Contact, Michael T. Heneka (michael.heneka@ukbonn.de).

This study did not generate new unique reagents.

### EXPERIMENTAL MODELS AND SUBJECT DETAILS

#### Animals

All animals used for cell isolation were treated according to the legal and ethical requirements of the University of Bonn – Medical Center (Germany). Mouse breeding and husbandry were approved by the veterinary office (Bonn, Germany) according to the German animal welfare act. The procedures complied with the guidelines of animal welfare as laid down by the German Research Council (DFG). For this study brains of P0–P2, mixed gender C57BL/6WT (purchased from Charles River Laboratories Inc.) and ASC-deficient (purchased from Millenium Pharmaceuticals) mice were used.

### METHOD DETAILS

#### Cell Culture

Primary microglia were isolated according to the method of Giulian and Baker (1986). After removing the meninges, cells were separated using mechanical shearing and 0,25% trypsin (GIBCO by lifeTechnologies™). Subsequently, cells were transferred into Poly-L-Lysine (PLL) (Sigma-Aldrich) coated T75 culture flasks (Greiner Bio-One) and cultured under standard conditions at 37°C and 5% CO_2_ (1-2 brains per flask). On the next day, flasks were washed three times with Dulbecco’s Phosphate Buffered Saline (DPBS) (GIBCO) and cultured for an additional 7-10 days in Dulbecco’s modified Eagle’s medium (DMEM) (GIBCO) containing 10% heat-inactivated Fetal Bovine Serum (iFBS) (GIBCO), 1% Penicillin/Streptomycin (P/S) (GIBCO) and 1 mL of filtered L929 cell supernatant as a source for growth factors. Cultures were regularly checked for loosely attached mature microglia. Finally, microglia were shaken off from the astrocyte monolayer after 7-10 days followed by two more shake off cycles every second to third day.

#### Preparation of Recombinant ASC

Full-length human ASC, followed by a TEV protease cleavage site and mCherry, was cloned in NdeI/XhoI sites of a pET-23a expression vector providing a C-terminal hexa-histidine tag. This construct was transformed and expressed in *E. coli* cells (strain BL21(DE3)) by growing the culture at 37°C to an OD_600_ of 0.8 and induced with 0.1 mM isopropyl β-D-1-thiogalactopyranoside for 4 h at 37°C. Cells were collected by centrifugation and lysed by sonication in a lysis buffer A containing 20 mM Tris (pH 8.0), 500 mM NaCl and 5 mM imidazole. Cell lysates were then centrifuged at 20,000 x g for 30 min and the pellet was dissolved in buffer A supplemented with 2 M Gdn-HCl for 1 h at 4°C. Subsequently, the suspension was again centrifuged at 20,000 x g for 30 min and the supernatant was dialysed against buffer A O/N at 4°C, while continuously stirring. On the next day, the sample was centrifuged as described above and the supernatant was administered to a pre-equilibrated HisTrap™ column using an Äkta Prime FPLC system (GE Healthcare). The column was washed with 10 column volumes of lysis buffer A and the protein was eluted in the same buffer supplemented with 200 mM imidazole. Subsequently, the purified protein was dialysed against buffer B containing 20 mM Tris (pH 8.0) and 300 mM NaCl O/N at 4°C, while continuously stirring. Finally, endotoxin concentration was controlled using the Pierce™ LAL Chromogenic Endotoxin Quantification Kit (Thermo Fischer Scientific) according to the manufacturers protocol. In order to separate soluble ASC monomers from insoluble aggregated forms the solution was centrifuged for 30 min at 100.000 x g in a TLA-120.2 rotor or equivalent in a Beckman Optima TLX benchtop ultracentrifuge. To induce fibrillation the ASC-containing solution was transformed to LoBind Tubes (Eppendorf) and incubated for 1 h at 37°C. The final concentration of ASC was quantified by NanoDrop using the extinction coefficient ε = 61.31. ASC fibrils were kept at 4°C for no longer than 3 weeks.

In addition to WT ASC, a mutated ASC was generated, carrying mutations in the PYD-PYD assembly interface (K21E, K22E, K26E) (Lu et al., 2014) and in the caspase-recruitment domain (CARD) (D134R, Y187E) (Li et al., 2018). Mutated ASC was generated following the same procedure as WT ASC.

#### Preparation of Amyloid β

Amyloid β (Aβ) protein (1-42) was ordered 1,1,1,3,3,3-Hexafluoro-2-propanol (HFIP)-treated (Bachem AG), dissolved in sterile Dulbecco’s phosphate buffered saline (DPBS) (GIBCO) and stored at −80°C (Götz et al., 2001). As a working concentration, 5 μM was used for cytotoxicity and cell viability assays, immunoblotting and ELISA.

For phagocytosis and degradation assays as well as for immunocytochemistry (ICC) FAM-labeled Aβ_1–42_ (Peptide Specialty Laboratories GmbH) was solved in 40 mM NaOH at 4 mg/ml, diluted in Tris-HCL (pH 7.4) to 1 mg/mL (221 mM), incubated for 1 day at 37°C and finally stored at −80°C.

#### Building ASC-Aβ Composites

Fibrillary ASC (1.75 mM) and Aβ_1-42_ monomers (5 μM) were incubated in serum-free DMEM containing 1% P/S and 1% N-2 supplement in LoBind Tubes (Eppendorf) at 37°C, O/N. The same culture procedure was applied to single protein treatments and the volume equal buffer controls.

#### Protein Determination

To determine protein concentrations of cell lysates, a bicinchoninic acid assay was performed using Pierce™ BCA Protein assay kit (Thermo Fischer Scientific) according to the manufacturer’s protocol.

#### Co-Immunoprecipitation (Co-IP)

For Co-IP experiments, 50 μL of SureBeads™ Protein G Magnetic beads (Bio-Rad Laboratories) were magnetized and washed with PBS (Dulbecco) supplemented with 0.1% Tween-20 (PBST) for three times. Thereafter, beads were incubated with either 2 μg of rabbit anti-ASC (clone AL177, AdipoGen) or 0.3 μg of mouse anti-Aβ (82E1) (IBL America) in a total volume of 200 μL PBST for 10 min at RT on a rotator. After that, beads were magnetized and washed three times with PBST. The antigen-containing solutions were added and incubated again for 1 h at RT on a rotator. For ASC-Aβ Co-IP’s 1.75 μM ASC and 5 μM Aβ were pre-incubated in serum-free medium O/N before co-culturing them with beads. For studying the intracellular bonding state of ASC and Aβ, ASC-deficient microglia lysates containing 35 μg total protein were added to a total volume of 200 μL PBST. Cell seeding, treatments and lysate collection were performed as explained in the “Immunoblotting (IB)” section. Antibody controls were incubated with 200 μL PBST in parallel. Subsequently, beads were magnetized and washed three times with PBST. To separate the antibody-bead binding, beads were resuspended in 40 μL loading buffer (106 mM Tris-HCL, 141 mM Tris base, 2% LDS, 10% glycerol, 0.51 mM EDTA (pH 8.5), 360 mM 1,4-Dithiothreit (DTT), and 5 mg/mL Orange G) and heated at 70°C, 600 rpm for 10 min. For immunoblotting, every vial was divided equally into two wells. Immunoblotting was then performed as described below.

#### Electron Microscopy

Fibrillary ASC (1.75 μM) and Aβ_1-42_ monomers (5 μM) were pre-incubated in buffer B (see above) O/N at 37°C. For negative staining electron microscopy, 4 μL of the protein sample was applied to a glow discharged copper grid and incubated for 1 min. Samples were washed three times by dipping the sample side into a drop of protein buffer before fluids were removed by the aid of a filter paper. Than samples were negatively stained by dipping them twice into a drop of 2% uranyl acetate following a 30 s incubation step and finally removing residual fluids using a filter paper. Afterward, the EM grid was air-dried and immediately processed. Samples were imaged using a JEOL JEM-2200FS 200 kV Transmission Electron Microscope (TEM) equipped with a CMOS-Camera (TemCam-F416, TVIPS).

#### Cytotoxicity and Cell Viability Assays

For viability and cytotoxicity experiments, primary microglia were seeded at a density of 7.5 × 10^4^ cells/well in 150 μL DMEM containing 1% P/S and 1% N-2 supplement (GIBCO) in a 96-well plate and allowed to attach O/N. After pre-simulation with 100 ng/mL lipopolysaccharide (LPS) (InvivoGen) for 3 h, wells were washed once with DMEM and treated with either 1.75 μM ASC, 5 μM Aβ, ASC-Aβ composites (containing 1.75 μM ASC and 5 μM Aβ) or its buffer controls (20 mM Tris (pH 8.0) and 300 mM NaCl (“buffer B”) for ASC and DPBS for Aβ) for 12 and 24 h. Subsequently, LDH release was measured using 50 μL supernatant and a cytotoxicity detection kit (Roche) according to the manufacturer’s protocol. The reaction was stopped with 1 N HCl and absorbance was measured at 490 and 680 nm using a microplate reader (Infinite M200; Tecan).

To determine cell viability, the XTT Cell Viability Kit (Cell Signaling Technology®) was used according to the manufacturer’s protocol. In brief, 150 μL phenol red-free DMEM (GIBCO) per well was mixed with 50 μL of XTT Reagent and 1 μL Electron Coupling Solution and added to the microglia. After 1 h the absorbance was measured at 450 nm with a TECAN microplate reader.

#### Immunoblotting (IB)

Primary microglia were seeded at a density of 1.5 × 10^6^ cells/well in 2 mL serum free DMEM in a 6-well plate. After pre-stimulating the microglia with 100 ng/mL LPS, cells were treated as mentioned in the “Cytotoxicity and Cell Viability Assays” section above. After 12 h of treatment, supernatants were collected, centrifuged at 15.000 x g for 5 min to remove cell debris, and stored at −20°C for protein precipitation.

For protein precipitation (Scheiblich et al., 2017), 500 mL methanol and 125 mL chloroform were added to 500 mL supernatant and vortex vigorously. Supernatants and used solutions were therefor kept on ice continuously. After 5 min centrifugation at 15,000 x g at 4°C the upper aqueous phase was removed carefully and again 500 mL ice-cold methanol were added to the remaining liquid. Samples were then vortexed vigorously and repeatedly centrifuged for 5 min at 13,000 x g at 4°C. Supernatants were removed and pellets were dried for 5 min in a vacuum dryer. The pellets were then resuspended in 10 μL 2 X loading buffer (see above) and denaturated (see below). Subsequently, samples were subjected to western blot analysis.

For lysate collection, cells were scraped of the well plate, centrifuged at 15,000 x g for 5 min and pellets were lysed using 1 X ristocetin-induced platelet agglutination (RIPA) buffer (25 mM Tris-HCl (pH 7.5), 150 mM NaCl, 0.5% sodium desoxycholate, 1% NP-40, and 0.1% SDS) supplemented with 1 X Protease/Phosphatase Inhibitor Cocktail (Cell Signaling Technology®). Cell lysates and precipitated supernatants were denaturated in loading buffer at 95°C and 360 rpm for 5 min in a thermo cycler. Samples were separated on a NuPAGE® 4%–12% Bis-Tris Gel (Invitrogen) in NuPAGE MES or MOPS SDS Running Buffer (NP0002) depending on the size of the respective protein of interest. The Trans-Blot® Turbo™ Transfer System (Bio-Rad Laboratories) was used to blot the proteins on a 0.2 μm nitrocellulose membrane (Trans-Blot® Turbo™ Transfer Pack, Bio-Rad Laboratories). Thereafter, membranes were blocked with 3% fatty acid-free bovine serum albumin (BSA) (Millipore) in Tris-buffered saline supplemented with Tween-20 (TBST) (10 mM Tris-HCl, 150 mM NaCl, 0.05% Tween-20, pH 8.0) for 1 h at RT, followed by incubation with the primary antibodies mouse anti-NLRP3 (1:1000, AdipoGen), rat anti-caspase-1 (1:1000, clone 4B4, Genentech), rabbit anti-ASC (1:1000; clone AL177, AdipoGen), rabbit anti-GSDMD (1:1000, Abcam), goat anti-TREM2 (1:1000, GeneTex), mouse anti-Aβ (82E1) (1:1000, IBL America), rabbit-anti-IL-1β (1:500, GeneTex), rabbit anti-GAPDH (1:1000, Sigma-Aldrich) and rabbit anti-β-actin (1:1000, Cell Signaling Technology®), O/N at 4°C, respectively. On the next day, membranes were washed three times in TBST and incubated with the respective secondary IRDye® IgG (H + L) antibodies (1:10 000, LI-COR Biotechnology) for 1 h at RT. Proteins were then visualized with the Odyssey Fc or CLx Imaging System (LI-COR Biosciences) and quantified using Image Studio (LI-COR Biosciences).

#### Measurement of Cytokine Secretion

Primary microglia were treated as described above in the “Cytotoxicity and Cell Viability Assays” section. Microglial IL-1β secretion was measured in cell supernatants using the mouse IL-1β/IL-1F2 DuoSet ELISA (R&D Systems) according to the manufacturer’s protocols. The reaction was terminated by adding 2 N H_2_SO_4_, and the optical density was measured at OD_450_ with a microplate reader. To determine cytokine concentrations, values were interpolated into the standard curve by linear regression using GraphPad Prism 7 (GraphPad Software).

For Toll-like receptor (TLR) neutralization experiments, cells were co-treated with anti-mouse TLR4/MD-2 Complex (clone: MTS510, Invitrogen), Mab-mTLR2 and anti-mouse TLR5 IgG (InvivoGen), as well as the mouse IgG Invitrogen) and rat IgG (Invitrogen) isotype controls at a final concentration of 5 μg/mL. For NLRP3 inflammasome inhibition, cells were co-treated with 1 μM MCC950 (CRID3) (Invivogen) or 100 nM IFM-2384 (IFM Therapeutics), respectively. A volume equal dimethylsulfoxid (DMSO) control, containing 0.005% DMSO was performed additionally.

#### Immunocytochemistry (ICC)

Primary microglia or ASC-deficient macrophages were seeded at a density of 2 × 10^5^ cells/well in 1 mL serum free DMEM in a 24-well plate containing PLL coated coverslips. Treatments were performed as described above. After 12 h, cells were washed once with PBS (Dulbecco) and fixed in 4% paraformaldehyde (PFA) dissolved in PBS for 15 min. For permeabilization, cells were washed three times with PBS containing 0.1% Triton X-100 (PTX) for 5 min. Thereafter, cells were blocked using 5% normal goat serum (Vector Laboratories) in PTX for 20 min and primary antibodies were added for another 30 min. To check for ASC speck formation, the rabbit anti-ASC (1:250; clone AL177, AdipoGen) or mouse-specific rabbit anti-ASC/TMS1 (1:250, D2W8U, Cell Signaling Technology®) and rat anti-CD11b (1:250; Serotec by Bio-Rad) were used. After three more washing steps in PTX, the secondary antibodies goat anti-rabbit (1:250; Invitrogen) and goat anti-rat (1:250; Invitrogen) were applied for 30 min followed by three washing steps. 4′,6-Diamidino-2′-phenylindol-dihydrochloride (DAPI) was used as a counterstain at 0.1 mg/mL for 20 min in PBS before coverslips were mounted. Images were taken using a 40 X or 60 X objective. To visualize caspase-1 activity, we used the FAM-FLICA® Caspase-1 Assay Kit ImmunoChemistry Technologies LLC) according to the manufacturer’s protocol. All images were acquired using a Nikon Eclipse Ti fluorescence 2 X microscope (Nikon). Image processing was accomplished using NIS-elements 4 (Nikon) and Fiji ImageJ (Wayne Rusband; National Institute of Health).

#### Phagocytosis and Degradation of Amyloid β

Primary microglia were seeded at 3.5 × 10^5^ cells/well in 1 mL serum free DMEM in a 24-well plate. Previously, 0.5 μM FAM-labeled Aβ (Fl-Aβ) (1-42) (Peptide Specialty Laboratories GmbH (PSL)) and 0.66 μM ASC were co-incubated in DMEM and kept in an incubator under standard conditions. For phagocytosis experiments, microglia were treated with either 0.5 μM FAM-Aβ or ASC-FAM-Aβ composites also containing 0.5 μM FAM-Aβ for 15, 30 and 60 min. For cell collection, supernatants were discarded, cells were washed with DPBS (GIBCO) and detached using 0.5% Trypsin-EDTA (GIBCO). Collected cells then were centrifuged at 300 x g for 5 min at 4°C. Supernatants were again discarded and cells were blocked in 50% iFBS diluted in DPBS for 10 min on ice. Subsequently, cells were centrifuged and pellets were resuspended in DPBS containing 2% iFBS and APC/CD11b antibody (1:100, clone M1/70, BioLegend) for 30 min on ice. After another centrifugation step, pellets were resuspended in DBPS containing 2% iFBS, and measured using the BD FACSCanto™ II Flow Cytometer. Detailed analysis was performed using FlowJo (FlowJo LLC/Becton Dickinson & Company).

For Aβ degradation experiments, microglia were exposed to FAM-labeled Aβ or ASC-FAM-Aβ composites concentrated as explained above for 1 h. Microglia were washed three times in PBS and subsequently incubated for 0, 1, 2, 3 or 4 h in fresh, FAM-Aβ-free or FAM-Aβ-ASC-composite-free medium. Thereafter, cells were collected and stained as described above. Degradation after 0, 1, 2, 3, and 4 h was measured using the BD FACSCanto™ II Flow Cytometer. Detailed analysis was performed using FlowJo.

### QUANTIFICATION AND STATISTICAL ANALYSIS

Data evaluation was performed using Graph Pad Prism 7 (GraphPad Software). Data are presented as mean ± SEM in all displayed diagrams. Every dataset is accrued from at least three independent experiments (n), containing at least two to three replicates (N). Each dataset was analyzed for Gaussian distribution. In case of passing the normality test, one-way ANOVA or tow-way ANOVA, for grouped datasets, were performed followed by a post hoc analysis with a Tukey test. Otherwise, non-parametric data was analyzed using the Kruskal-Wallis test combined with a Dunn’s post hoc test. When only two groups were statistically analyzed, a t test was performed. For non-parametric data the Mann-Whitney test was applied. For each individual experiment the statistical details can be found in the corresponding figure legends. Levels of significance are indicated as *p < 0.05; **p < 0.01; ***p < 0.001; ****p < 0.0001.

### DATA AND CODE AVAILABILITY

This study did not generate/analyze any unique datasets or codes.

## Supplementary Material

1

## Figures and Tables

**Table:** KEY RESOURCES TABLE

REAGENT or RESOURCE	SOURCE	IDENTIFIER
Antibodies		
Rabbit anti-Asc (AL177) antibody	AdipoGen	Cat# AG-25B-0006; RRID: AB_2490440
Rat anti-Caspase-1 (clone 4B4) antibody	Genentech	Cat# CASP-1(mu):4175
Rabbit anti-GAPDH antibody	Sigma-Aldrich	Cat# G9545; RRID: AB_796208
Rabbit anti-GSDMD antibody [EPR19828]	Abcam	Cat# ab209845; RRID: AB_2783550
Mouse anti-NLRP3/NALP3 (Cryo-2) antibody	AdipoGen	Cat# AG-20B-0014-C100; RRID: AB_2490202
APC rat anti-mouse/human CD11b antibody	Bio Legend	Cat# 101212; RRID: AB_312795
Mouse specific rabbit anti-ASC/TMS1 (D2W8U) antibody	Cell Signaling Technology	Cat# 67824; RRID: AB_2799736
Rabbit anti-β-actin antibody	Cell Signaling Technology	Cat# 4967; RRID: AB_330288
Goat anti-rabbit-AlexaFluor488 (H+L) antibody	Thermo Fisher Scientific	Cat# A-11008, RRID: AB_143165
Goat anti-rat-AlexaFluor594 antibody	Invitrogen	Cat# A11007; RRID: AB_141374
Rabbit anti-IL-1β antibody	GeneTex	Cat# GTX74034; RRID: AB_378141
IRDye® 680LT donkey anti-mouse IgG (H+L) antibody	LI-COR Biotechnology	Cat# 926-68022; RRID: AB_10715072
IRDye® 800CW goat anti-rat IgG (H+L) antibody	LI-COR Biotechnology	Cat# 926-32219; RRID: AB_1850025
Mouse anti-Aβ (82E1) antibody	IBL America	Cat# 10323; RRID: AB_1630806
Mouse IgG Isotype Control antibody	Thermo Fisher Scientific	Cat# 10400C; RRID: AB_2532980
Mouse TLR2 neutralizing antibody (C9A12)	InvivoGen	Cat# mabg-mtlr2; RRID: AB_11125339
Mouse TLR5 neutralizing antibody (Q23D11)	InvivoGen	Cat# mabg-mtlr5; RRID: AB_11124926
Rat anti-mouse CD11b antibody	Serotec by Bio-Rad	Cat# MCA711; RRID: AB_321292
Rat IgG Isotype Control antibody	Thermo Fisher Scientific	Cat# 10700; RRID: AB_2610661
TLR4/MD-2 Complex Monoclonal Antibody (MTS510)	Invitrogen	Cat# 14-9924-82; RRID: AB_468617
Goat anti-Trem2 antibody	GeneTex	Cat# GTX47596; RRID: AB_10618011
Chemicals, Peptides, and Recombinant Proteins		
Ampicillin sodium salt	Carl Roth	Cat# K029.1
4’,6-Diamidino-2′-phenylindol-dihydrochloride (DAPI)	Thermo Fisher Scientific	Cat# 62247
Adenosine 5′-triphosphate disodium salt hydrate	Sigma-Aldrich	Cat# A2383
Amyloid β-Protein (1-42) (HFIP-treated)	Bachem AG	Cat# 4090148
Bovine Serum Albumin - Fraction V	Rockland Immunochemicals, Inc.	Cat# BSA-1000
FAM-labeled Amyloid β	Peptide Specialty Laboratories GmbH (PSL)	ID# CEM112812FL
Fetal Bovine Serum	LIFE Technologies	Cat# 10270106
Guanidine hydrochloride	Carl Roth	Cat# 6069.3
HisTrap™ FF crude	GE Healthcare	Cat# 17528601
IFM-2384	IFM Therapeutics	gift from IFM
Lipopolysaccharide from *Escherichia coli* K12	InvivoGen	Cat# tlrl-eklps
MCC950 (CRID3)	InvivoGen	Cat# inh-mcc
N2-Supplement	GIBCO by Thermo Fisher Scientific	Cat# 17502048
NdeI	New England Biolabs	Cat# R0111S
Normal Goat Serum	Abcam	Cat# ab7481
NuPAGE® 4-12% Bis-Tris gel	Invitrogen	Cat# NP0323BOX
NuPAGE MES SDS Running Buffer (20X)	Thermo Fisher Scientific	Cat# NP0002
Orange G	Carl Roth	Cat# 0318.2
Paraformaldehyde	Sigma-Aldrich	Cat# P6148
pET23a	Novagen	Cat# 69745
pET23a-ASC-tev-mCherry	Venegas et al., 2017	N/A
Poly-L-Lysine Hybridomide	Sigma-Aldrich	Cat# P1524
Protease/Phosphatase Inhibitor Cocktail (100X)	Cell Signaling Technology	Cat# 5872
Protein LoBind Tubes	Eppendorf	0030108116
Q5 High Fidelity DNA polymerase	New England Biolabs	Cat# M0491S
SureBeadsTM Protein G Magnetic beads	Bio-Rad Laboratories	Cat# 1614023
T4 DNA Ligase	New England Biolabs	Cat# M0202S
Trans-Blot® Turbo Mini Nitrocellulose Transfer Packs	Bio-Rad Laboratories	Cat# 1704158
Critical Commercial Assays		
Cytotoxicity Detection Kit (LDH)	Roche	Cat# 11644793001
FAM-FLICA® Caspase-1 Assay Kit	ImmunoChemistry Technologies	Cat# 98
Mouse IL-1 beta/IL-1 F2 DuoSet ELISA	R&D Systems	Cat# DY401
Pierce™ BCA Protein Assay kit	Thermo Fischer Scientific	Cat# 23225
Pierce™ LAL Chromogenic Endotoxin Quantitation Kit	Thermo Fischer Scientific	Cat# 88282
QIAprep Spin Miniprep Kit	QIAGEN	Cat# 27104
QIAquick Gel Extraction Kit	QIAGEN	Cat# 28704
QIAquick PCR Purification Kit	QIAGEN	Cat# 28104
XTT Cell Viability Kit	Cell Signaling Technology	Cat# 9095
Experimental Models		
NCTC clone 929 (L929 cells), strain: C3H/An	ATCC	Cat# CCL-1; RRID: CVCL_0462
Primary microglia isolated from C57BL/6 mice	Charles River Laboratories	RRID: IMSR_JAX:000664
Primary microglia isolated from C57BL/6 ASC^−/−^ mice	Millenium Pharmaceuticals	N/A
*E. coli BL21 (DE3), genotype: F– ompT hsdSB (rB–, mB–) gal dcm (DE3)*	Merck	Cat#69450.
Software and Algorithms		
FACSDIVA software	Becton Dickinson	N/A
Fiji ImageJ	Wayne Rusband	v2.0.0-rc-69/1.52n
FlowJo	FlowJo, LLC	V3.05470
Graph Pad Prism	GraphPad Software Inc.	v7.0e
Image Studio	LI-COR Biosciences	v5.2
NIS-Elements	Nikon	v4.0
Other		
Avanti J265 XP centrifuge	Beckmann Coulter	equipment
ÄKTAprime plus FPLC system	GE Healthcare	equipment
BD FACSCanto™ II	BD Biosciences	equipment
Eppendorf BioPhotometer D30	Eppendorf	equipment
Infinite M200 Pro	TECAN	equipment
JA-25.50 Fixed-Angle Rotor	Beckmann Coulter	equipment
JEOL JEM-2200FS Field Emission Transmission Electron Microscope equipped with a CMOS-Camera	JEOL GmbH	equipment
JLA-8.1000 Fixed-Angle Rotor	Beckmann Coulter	equipment
JULABO 5 water bath	JULABO GmbH	equipment
Lab 850 pH-Meter	Schott Instruments	equipment
Multitron pro	Infors HT	equipment
NanoDrop 2000c Spectrophotometer	Thermo Scientific	equipment
Nikon Eclipse Ti Fluorescence Microscope	Nikon	equipment
ODYSSEY CLx Imaging System	LI-COR Biotechnology	equipment
Optima TLX ultracentrifuge	Beckmann Coulter	equipment
TLA-120.2 Fixed-Angle Rotor	Beckmann Coulter	equipment
Trans-Blot® Turbo™ Transfer System	Bio-Rad Laboratories	equipment
Vibra-Cell ultrasonic liquid processor	Sonics	equipment

## Highlights

- Exogenous ASC can be incorporated by the NLRP3 inflammasome of the recipient microglia
- Aβ binding to ASC fibrils amplifies NLRP3 inflammasome activity
- Microglia exposed to ASC-Aβ composites undergo pyroptotic cell death
- Intra- and extracellular ASC-Aβ binding impairs Aβ clearance by microglia
