# Supplementary material for: β-Amyloid Clustering around ASC Fibrils Boosts Its Toxicity in Microglia
Source: Cell Rep. Author manuscript; Available in PMC 2022 Jan 5. (PMC8729885; doi:10.1016/j.celrep.2020.02.025)
Supplement: 1 [file NIHMS1686483-supplement-1.pdf]

Cell Reports, Volume *30*

Supplemental Information

β-Amyloid Clustering around ASC Fibrils

Boosts Its Toxicity in Microglia

Lea L. Friker, Hannah Scheiblich, Inga V. Hochheiser, Rebecca Brinkschulte, Dietmar Riedel, Eicke Latz, Matthias Geyer, and Michael T. Heneka

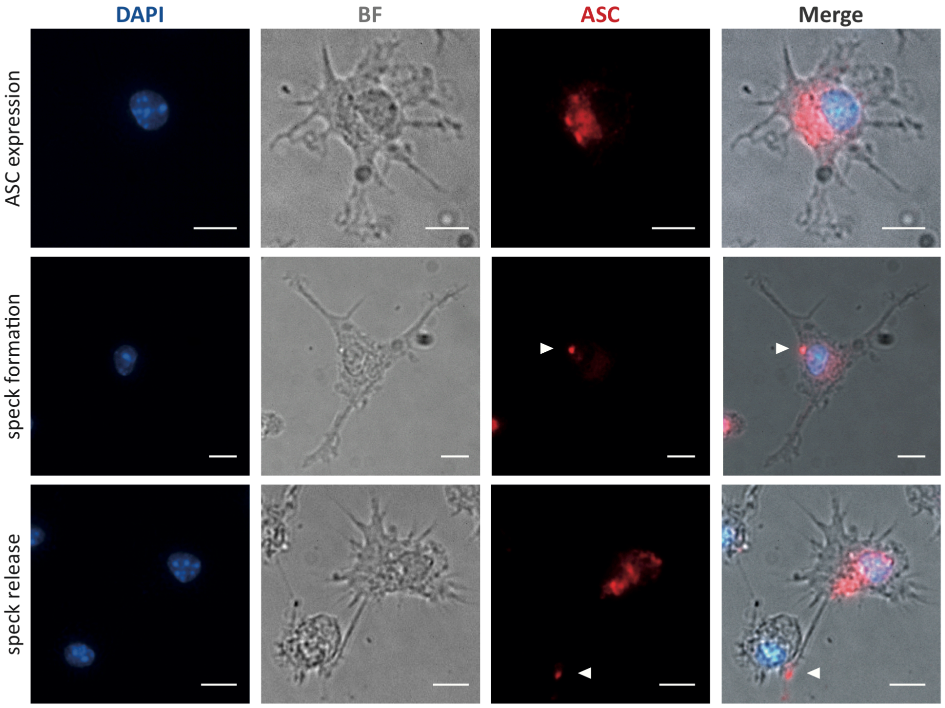

Fig. S1 | Exogenous ASC induces expression and specking of endogenous ASC. Related to Fig. 2 and Fig. 3. Immunocytochemical staining of ASC using a mouse-specific antibody (D2W8U). Top row: ASC expression, middle row: ASC speck formation, bottom row: ASC speck release. Bright-field (BF). Images were taken at 60 X magnification. Scale bar, 10 µm.

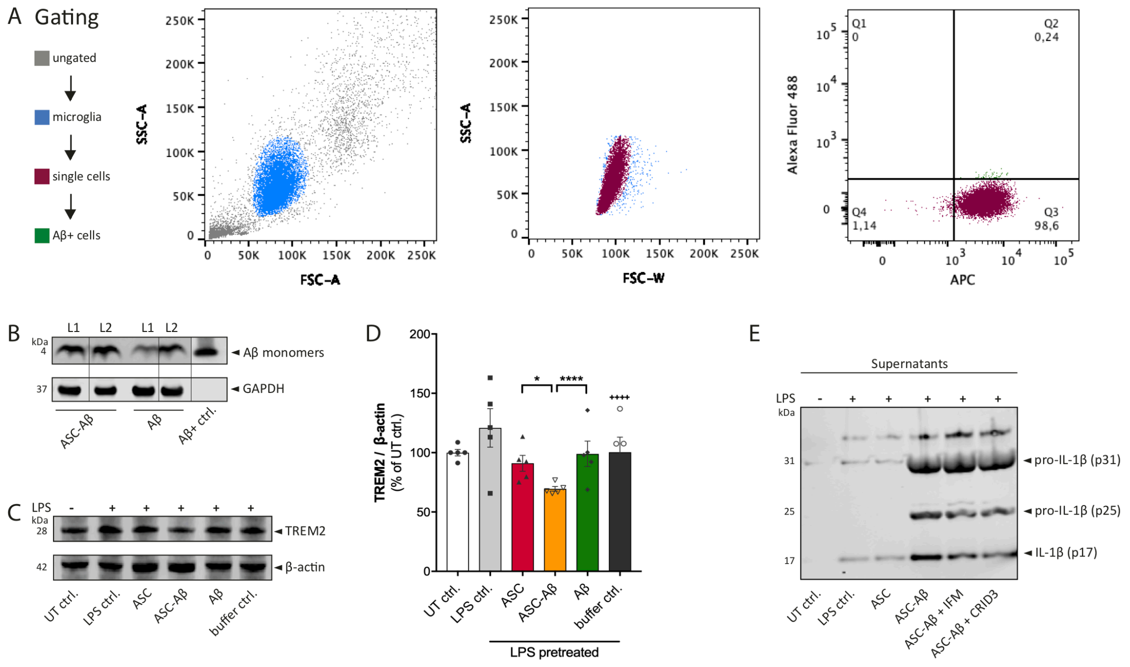

Fig. S2 | FACS gating. Treatment with ASC-Aβ composites reduces TREM2 expression in primary microglia. Related to Fig. 3 and Fig. 5.

All experiments displayed were performed using primary WT microglia. **A** FACS gating. Ungated (grey) → CD11b+ population, considered microglia (blue) → singlets (purple) → Aβ+ single microglia in Q2 (green). **B** Western blot of microglia cell lysates primed for 3 h with 100 ng/ml LPS and exposed to Aβ alone or ASC-Aβ composites for 12 h, stained for Aβ (82E1). **C, D** TREM2 expression detected in microglia lysates by western blot after 12 h of treatment post LPS-priming. Data were collected from two (**B**) or five (**C, D**) independent experiments (n = 2, n = 5). (**E**) Representative immunoblot for IL-1β in microglia supernatants treated with ASC-Aβ without or with co-application of the NLRP3 inflammasome inhibitors IFM-2384 or CRID3. All graphs are presented as mean ± SEM and were analysed by unpaired t-test. Levels of significance are indicated as *$p < 0.05$; **$p < 0.01$; ***$p < 0.001$; ****$p < 0.0001$. Star-symbol indicates significance between groups connected by lines; plus-symbol indicates significance between ASC-Aβ composites and volume equal buffer ctrl. treated groups.
